# Clinicians’ experiences implementing an advance care planning pathway in two Canadian provinces: a qualitative study

**DOI:** 10.1186/s12875-024-02468-4

**Published:** 2024-06-15

**Authors:** Julie Stevens, Dawn Elston, Amy Tan, Doris Barwich, Rachel Zoe Carter, Diana Cochrane, Nicole Frenette, Michelle Howard

**Affiliations:** 1 End-of-Life Care Research Group, Vrije Universiteit Brussel, Laarkbeeklaan 103, Brussels, Belgium; 2 Department of Family Medicine, McMaster University, 1280 Main Street West, Hamilton, ON Canada; 3 Department of Medicine, Faculty of Medicine, University of British Columbia, 2329 West Mall, Vancouver, BC Canada; 4 BC Centre for Palliative Care, 300 – 601 Sixth St., New Westminster, BC Canada; 5 Department of Family Medicine, University of Calgary, 2500 University Drive NW, Calgary, AB Canada

**Keywords:** Advance care planning, Primary care, Internal medicine, Interview study, Focus group study

## Abstract

**Background:**

Advance care planning (ACP) is a process which enables patients to communicate wishes, values, fears, and preferences for future medical care. Despite patient interest in ACP, the frequency of discussions remains low. Barriers to ACP may be mitigated by involving non-physician clinic staff, preparing patients ahead of visits, and using tools to structure visits. An ACP care pathway incorporating these principles was implemented in longitudinal generalist outpatient care, including primary care/family medicine and general internal medicine, in two Canadian provinces. This study aims to understand clinician experiences implementing the pathway.

**Methods:**

The pathway was implemented in one family practice in Alberta, two family practices in British Columbia (BC), and one BC internal medicine outpatient clinic. Physicians and allied health professionals delivered structured pathway visits based on the Serious Illness Conversation Guide. Twelve physicians and one social worker participated in interviews or focus groups at the end of the study period. Qualitative data were coded inductively using an iterative approach, with regular meetings between coders.

**Results:**

Clinicians described experiences with the ACP care pathway, impact at the clinician level, and impact at the patient level. Within each domain, clinicians described barriers and facilitators experienced during implementation. Clinicians also reflected candidly about potential for future implementation and the sustainability of the pathway.

**Conclusions:**

While the pathway was implemented slightly differently between provinces, core experiences were that implementation of the pathway, and integration with current practice, were feasible. Across settings, similar themes recurred regarding usefulness of the pathway structure and its tools, impact on clinician confidence and interactions with patients, teamwork and task delegation, compatibility with existing workflow, and patient preparation and readiness. Clinicians were supportive of ACP and of the pathway.

**Trial registration:**

The study was prospectively registered with clinicaltrials.gov (NCT03508557). Registered April 25, 2018. https://classic.clinicaltrials.gov/ct2/show/NCT03508557.

**Supplementary Information:**

The online version contains supplementary material available at 10.1186/s12875-024-02468-4.

## Background

Older adults and patients with a life-limiting illness benefit from making their wishes for care known prior to an acute health event, which may leave them unable to communicate those wishes or make medical decisions. The process of communicating wishes, values, fears, and preferences for future medical care between the patient, their loved ones, and multidisciplinary health care professionals, with the goal of helping to ensure that patients receive medical care consistent with their values, is known as advance care planning (ACP) [1, 2]. Engaging in ACP is associated with greater concordance between care preferences and care received, higher quality of patient-clinician communication [3], higher quality of care at the end of life [4], greater sense of control for the patient [5], and reduced distress for substitute decisions makers (SDMs) [6]. Crucial to the ACP process are a timely start and iterative conversations with health care providers [7, 8]. Outpatient care settings, such as primary care, have been proposed as an ideal setting to initiate and facilitate ACP. The longitudinal and trusting patient-provider relationship enables these iterative conversations [9–11], at a time when the patient’s health is relatively stable [12, 13]. 

However, despite older adults’ and patients’ interest in discussing ACP, conversations in health care settings occur infrequently [9, 14, 15]. In Canada, a survey of elderly hospitalized patients showed that most had thought about end-of-life care, but only half of patients who discussed their wishes had done so with any member of a health care team [16]. The frequency of discussions, including with family physicians, remains low [17, 18]. Barriers also persist in outpatient care settings; in a national survey of Canadian primary care providers, engagement in ACP was low despite high willingness and confidence [19]. A lack of time is a prominent barrier at the clinician level [20–22]. The involvement of non-physician clinic staff, such as nurses, may reduce time-related barriers through delegation of tasks [23]. In Canada, primary care clinicians are supportive of involving non-physician clinicians in ACP [19]. This represents a potentially underutilized resource, as well as a knowledge gap regarding the role of allied health professionals for ACP in (Canadian) longitudinal generalist outpatient care settings.

Combining tools which assure clinicians know what to discuss, with information provided to patients and family prior to the clinic visit, can also increase the efficiency of visits themselves [23, 24]. The Serious Illness Conversation Guide (SICG) [24] is one such tool. As a component of the Serious Illness Care Program (SICP) communication intervention, it provides a structured approach for ACP topics [25]. The SICP may promote more and better conversations about patient care wishes [26], by supporting physicians and non-physician clinicians to implement timely conversations into their practice routines [27]. Evaluations of such interventions in longitudinal generalist outpatient care are still limited; a prior study evaluated Canadian primary care clinician experiences implementing the SICP and found that a more systematic process of implementation may be needed [10]. 

There is a need to evaluate an approach that combines principles of interprofessional collaboration within the clinic, with the structure of the SICG tool. To this end, a multi-faceted ACP pathway was implemented in longitudinal generalist outpatient care clinics, including primary care and general internal medicine, in Alberta and British Columbia (BC), Canada. It is important to examine the implementation processes of this pathway, to ensure that it is workable and can be durably integrated into practice, by attending to how it interacts with the existing organization of care [28]. Normalization Process Theory (NPT) [29], an implementation science framework, is of use in exploring the context for implementation, whether the pathway is seen as relevant and important, and how new processes introduced by the pathway interact with existing processes in the clinic setting. The framework assesses sense-making (coherence), relational work (cognitive participation), operational work (collective action), and appraisal (reflexive monitoring), and served as the background for examining lived experience with the pathway in two Canadian provinces.

## Methods

### Aims

This study aims to explore the experiences of physicians and allied health professionals in two Canadian provinces (Alberta and British Columbia) with implementing the ACP pathway, using the NPT framework as the basis for interviews. The consolidated criteria for reporting qualitative research (COREQ) [30] were used to structure the report (Additional File 1).

The project to test the ACP care pathway was undertaken in Alberta and BC, Canada, from 2018 to 2020. We used a qualitative approach to describe the implementation in participating clinics. Participating clinics provided longitudinal outpatient generalist care of adults. One family practice in Alberta, two family practices in BC, and one BC internal medicine outpatient clinic participated in the project. General internal medicine, which is not a primary care setting in Canada, was considered legitimate to include alongside primary care clinics, as internal medicine clinics are designed to manage complicated illness and patients may have an established relationship with this setting.

### The ACP pathway

The ACP pathway is an intervention based on the Serious Illness Conversation Guide (SICG) [24]. Physicians and Allied health professionals first received SICG training.

The patient-facing portion of the pathway consisted of three steps (see Additional File 2 for more detailed information). Step 1 covered consent, research questionnaires, and ACP education, such as how to choose a SDM. Step 2 was an ACP education and values clarification session, including completion of the End of Life Values Best-Worst Scenario Online Tool with the patient. This tool allows patients to rank issues they find the most and least important, when considering medical treatment at the end of life [31, 32] (Additional file 3). Step 2 resulted in a Dear Doctor Letter (see Additional File 2) stating patient wishes, including a summary output from the online tool, which was also provided to the physician. In BC, steps 1 and 2 were combined into one visit with the research coordinator and a research nurse due to practical considerations. Patients were asked to bring someone who they would consider their SDM, if available, to the visit. In Alberta, step 2 was scheduled with an allied health professional during a second visit 2–6 weeks after step 1. Approximately 2–4 months after the first visit, patients met with the physician for step 3, which focused on finalizing and documenting ACP.

### Inclusion

Participating physicians who attended the SICG training identified eligible patients (≥ 60 years of age and/or at risk for health decline due to serious or life-limiting illness, according to a list of indicators such as the “Surprise Question”: Would the physician be surprised if the patient passed away within 2 years? ) from their electronic medical record (EMR). With patient approval, physicians provided patient contact information to a researcher, who contacted the patient to schedule a research appointment. For the qualitative study, physicians who expressed interest, completed the SICG training, and referred patients to the ACP pathway were interviewed.

### Data collection procedure

The study was stopped in Alberta at the start of the COVID-19 pandemic, with interviews and focus groups conducted until March 2020. Pathway meetings in BC were stopped in March 2020; interviews were conducted until October 2020. Interviews and focus groups were conducted by two co-authors (DC, MHLP; NF, MSW) and one additional interviewer, all of whom are female. Interviews were conducted in the clinic setting, or via telephone contact due to COVID-19. One physician (BC) was contacted for a member-checking interview during data analysis in July 2022. JS (female, MSc) and DE (female, MA) conducted the interview via video-conference and took extensive written notes. Interviewer background included research assistant, research coordinator, and doctoral researcher. Clinicians interviewed were aware of the project and of the interviewers’ reasons for doing the research.

Interviews and focus groups were semi-structured using an interview guide based on NPT as theoretical framework (Additional file 4). The interview guide followed general NPT questions, e.g. “How did the intervention affect the work of the practice?”, each with corresponding open questions and additional prompts.

In BC, nine physicians (including physicians who were unable to refer patients) were invited for interviews; five physicians participated, all of whom had referred patients. In Alberta, all participating physicians and the one participating social worker were interviewed.

### Analyses

Interviews were audio-recorded and transcribed verbatim. Two researchers (JS, DE) independently analysed the transcripts. Transcripts were first read multiple times to gain familiarity with the data. Although the use of an interview guide provided an initial structure to the topics within the transcripts, an inductive approach was used during coding, rather than strictly imposing the interview questions as a framework, so that themes could emerge organically. Codes and preliminary themes were compared after independent analyses of the first transcript, and a preliminary codebook with domains, themes, and sub-themes was established. The codebook was then used to independently code the remaining transcripts using NVivo 12 and Microsoft Excel software.

Regular meetings allowed the coders to iteratively update the codebook with newly-emerging codes; to generate new themes, adjust the naming, structure, and content of themes; and to resolve discrepancies through discussion. In the case of unresolved discrepancies, a third researcher (MH) was invited to arbitrate. The third researcher also checked the final coding framework. JS and DE researchers populated the framework with illustrative quotes; MH checked the relevance and clarity of the selected quotes.

## Results

In the three participating BC sites, three family physicians (1 female, 2 male) and two internal medicine physicians (1 female, 1 male) were interviewed one-on-one. In Alberta, three focus groups were conducted with a total of seven family physicians (3 male, 4 female), and one social worker (female) was interviewed individually. Interviews and focus groups lasted approximately 30 to 60 min.

We identified three overarching domains describing experiences with the ACP pathway, impact at the clinician level, and impact at the patient level. Within each domain, we identified subthemes as they related to facilitators and barriers experienced by physicians during implementation of the pathway. Some physician responses related to potential for future implementation and the sustainability of the pathway, outside the context of the current study. Physicians described these future considerations in relation to experiences with the pathway itself, as well as physician- and clinic-level impact (Table 1; See Table S1 in Additional File 5 for illustrative quotes).

**Table 1:** Domains, themes, and subthemes

Domain	Theme	Subtheme
Care Pathway	Facilitators	Documents, forms, and tools are helpful
		Sequential structure is easy to implement
	Barriers	Appointments: preparation, duration, and modality
		Negative patient experiences with the pathway
		Clinical care coordination
	Future sustainability	Adapt to emerging needs (Virtual care, COVID, …)
		Embed and normalize ACP
		Expand training
		Need for communication and bridging tools
		Broader health care system implications
Clinical Practice	Facilitators	Patient/SDM willingness, readiness, and preparation
		Positive impact on clinicians
		Positive impact on clinicians’ interaction with patients
	Barriers	Practical challenges to visits
		Patients/SDMs may not be ready
	Future sustainability	No billing codes
Teamwork	Facilitators	Social worker has the necessary skills for ACP and is a referral for more complicated conversations
		Awareness of staff
		Promotes teamwork and strengthens existing collaborative relationships
	Barriers	Availability of resources : staffing, structural barriers, team composition
		Unclear division of tasks
		Problems coordinating between visits
		Different service models do not support the pathway in its current form
	Future sustainability	Training other staff
		Who will be available after the study is over?
		Expanding visit 2
Work processes	Facilitators	Efficiency and integration with current workflow
		Recognition of inherent value of the intervention
		Benefits of recruitment strategy
	Barriers	Individual/practice-level barriers
		System-level barriers
	Future sustainability	Virtual vs. in-person visits for different purposes and populations
		Placing the pathway in context of health care system changes
		Tailoring to local context and adapting to individual clinic needs
Preparation	Facilitators	Pre-work for follow-up visits
		Patient-centered, ongoing conversation
	Barriers	Patient lack of buy-in
		Need for more clarity, time, discussion
		Difficulty translating goals into levels of care
Readiness	Facilitators	Promotes readiness to have goals-of-care discussions and complete documents
		SDM is aware of patient values and wishes
	Barriers	Difficult transition from thinking about values to documenting goals of care
		Lack of patient comfort and energy

### The care pathway

The first overarching domain refers to ease of use of the care pathway, such as the sequential structure with appointments and documents/tools. Clinicians evaluated the components of the pathway as clear, understandable, and useful. The sequential structure allowed easier referrals to ACP and Goals of Care discussions and supported existing practices, such as complex care visits.

Barriers to implementing the pathway included difficulty accommodating additional preparatory work, such as tracking documentation of ACP in the EMR. Some clinicians were uncertain how well the pathway would work as virtual visits, which were necessary during the COVID-19 pandemic. The pathway’s dependence on the use of specific ACP tools by the patient, could pose a barrier to coordination with clinical care.

Clinicians reflected on the potential to adapt the pathway to emerging needs, such as virtual care. The training component was foundational to the success of the pathway and physicians recommended expanding the training. Embedding and normalizing ACP within the practice culture was an important prerequisite; clinicians recommended this would include challenging perceptions of which health staff are responsible for ACP. Finally, clinicians suggested a need for bridging tools to facilitate transitions between visits, and communicate information with other physicians who may be in contact with patients involved in the pathway.

### Clinician impact

Clinicians discussed the impact of the pathway on domains related to their practice and interaction with patients, teamwork within the practice, and their work processes.

The domain of *clinical practice* refers to roles and responsibilities of the individual physician, and the clinical interactions between physician and patient. The pathway facilitated these interactions through the preparedness of patients and SDMs, who had a clear rationale for their visit. At the physician level, a script with tested, validated ways to talk about ACP improved confidence in the decision-making process. When family physicians felt confident and understood patients’ long-term goals, this positively affected their interactions with patients, leading to deeper conversations where ACP could be discussed comfortably. Barriers included practical challenges, such as a lack of time, cancelled appointments, and difficulty planning visits, resulting in lost momentum during the clinical process. When patients showed discomfort discussing the end of life, or a lack of readiness to choose an SDM, some clinicians may not have known how to move the needle on these difficult conversations. When clinicians felt that patients were not ready, they perceived themselves instead as “nagging” these patients into entering the pathway. It was also noted that in one jurisdiction, conversations such as those in the pathway did not fall under existing billing codes, possibly precluding clinicians from investing time in these conversations.

The *teamwork* domain refers to how the work of the pathway was allocated within the practice team, and how members of the team cooperated to implement it. When physicians were aware that allied health professionals had the skill set for ACP, they had greater willingness to refer patients for complicated ACP conversations. Staff awareness of and support for the care pathway in turn translated to practical support for physicians in their tasks. This division of tasks according to expertise enhanced existing collaborative relationships within the practice. The division of tasks among team members in the pathway was also a barrier. There were challenges where the allied health professional conducting some of the visits was external to the clinic as this was a new role that needed to be incorporated. Lack of clarity of roles in some situations hindered efficient teamwork. Due to the multiple individuals involved, one physician perceived that the patient became a “go-between” between the social worker and the physician. In considering future sustainability in the teamwork domain, clinicians proposed training and engaging the entire practice, including medical residents, to allow delegation of tasks according to practice resources. Some physicians questioned the long-term sustainability of including allied health after the conclusion of the study and considered how clinic staff roles might change to accommodate their absence. One proposed solution was integrating ACP care pathway visits within an existing pathway for complex care visits.

The *work processes* domain refers to how work was previously done and how new ways of working were integrated into the pathway. Impact was facilitated via compatibility between the pathway and current workflow, allowing integration with existing activities and the clinic scope of practice. This streamlined the ACP process; decision-making was more robust without requiring additional time. As clinicians recognized the inherent value of ACP, they noted that time spent on ACP conversations was time well spent. An additional facilitator emerged following the use of patient lists (via a query in the EMR) for study recruitment: clinicians suggested that identifying patients eligible for ACP may be more effective and less threatening to patients when it is framed as part of routine clinical practice. Barriers were considered at the level of clinicians and their practice, and the health system level. There was difficulty integrating the pathway into the current way of working when, for instance, pre-planning for visits created additional work for physicians who may otherwise have engaged in ACP “in the moment”. Physicians did not want to take time away from visits for other purposes, such as medical consultations; some suggested that ACP would need a separate conversation. However, fee-for-service models were seen as less compatible with this approach. Additionally, integration with the existing workflow was, for example, difficult for a physician in the internal medicine setting, who did not have a regular schedule in the clinic. Clinicians proposed flexibility in implementation to better integrate the pathway into their existing workflow. Many clinicians proposed recommendations for tailoring the pathway to the local context and adapting it to individual clinic needs. Additional consideration was given to future changes in the health system, such as in physician contracts and salaries, which may facilitate more ACP.

### Patient impact

Fewer responses by clinicians referred specifically to impact at the patient level. We distinguish between the impact on patient preparation and patient readiness.

*Preparation* refers to patients’ engaging with ACP, prior to the physician visit. Pre-work for follow-up visits, i.e., using tools to help patients align values with care goals, was a facilitator for this preparation, as patients were actively involved and became invested in the ACP process. Enhanced comfort and better communication between patient and SDM resulting from this pre-work contributed to preparation. As the pathway focused on promoting ongoing conversations in a patient-centered way, patients were seen to be able to build on knowledge they may already have. A lack of buy-in from patients or fluctuating willingness to participate was a prominent barrier to preparation, which could occur at different points in the pathway. Patients may also have needed more time and information to prepare for the physician visit. Some patients, such as those who were still relatively well, had not thought about resuscitation or had trouble imagining a time when they could not speak for themselves, impeding contemplation about possible goals of care.

*Readiness* refers to patients engaging with ACP during or following the visit with the physician. In addition to practical preparation for conversations regarding goals of care, clinicians felt the pathway conversations helped patients feel ready to engage with details of the ACP process and to discuss goals of care, and that SDM confidence increased. Difficulty transitioning from thinking about values to documenting concrete goals of care impeded patient readiness. Compared to having ACP conversations, completing goals of care documentation was more difficult for patients, and patients had less confidence in the end results of this documentation. Patient comfort and energy was a final potential barrier, resulting from a protracted process and long conversations with clinicians.

## Discussion

### Findings

This study presents the lived experiences of clinicians in two Canadian provinces implementing a novel ACP pathway within longitudinal generalist outpatient settings, including primary care/family medicine and general internal medicine.

Much attention was paid to elements of sense-making (NPT construct: coherence) and relational work (NPT construct: cognitive participation). In line with previous research, clinicians saw ACP as important [33], and as a legitimate part of their clinical work. The pathway introduced new tools, a standardized structure, and a new role for allied health, which differed from existing ad-hoc approaches. However, several clinicians reported lack of buy-in from patients, a lack of follow-up after patients entered the pathway, or a lack of readiness to participate from the SDM. These barriers at different points, reported by clinicians, correspond to patient-reported barriers described in prior research such as the perceived importance or relevance of ACP [34], or distrust towards formal documentation of ACP [35]. This highlights an invaluable contribution to sense-making work from the patient and SDM. Acceptability and success of the pathway relies on patients feeling ready for, or wishing to engage with, conversations about serious illness and the end of life. This should be considered in future initiatives to implement similar pathways in clinics. Fostering experiences of shared decision-making throughout the patient’s life could ease the transition to making decisions about care in serious illness and at the end of life [36]. 

Contributions to teamwork within the clinic and impact on work processes reflect operational work done to enact the pathway (NPT construct: collective action). Physicians felt that allied health professionals had the knowledge and skills to broach ACP and valued their support, while allied health also acknowledged which tasks were better suited for the physician. Involvement of other clinic team members varied from being mostly uninvolved, to staff actively supporting clinicians, such as by bringing documentation to them. This finding lends practice-based evidence to prior survey findings that primary care physicians find it acceptable for non-physician staff to be involved in ACP [19] and suggests a team-based approach to ACP in the clinic setting is desirable and feasible. Positive and trusting interprofessional relationships, and clarity of roles and responsibilities, are facilitators to implementation of interventions into primary care [37]. Although staffing availability may differ depending on context, the role division in the care pathway was implemented to a degree that suggests the model is sustainable and could be expanded further within the clinic team for the future. Spontaneous recommendations to include other clinic staff, such as medical residents and learners, for training and implementation in the future, are especially encouraging for sustainable implementation. Facilitating consistent communication between all team members will be important to ensure momentum is maintained in the ACP process.

There was variable feedback about the feasibility of integrating the pathway with existing practices. A recurring barrier was a lack of time to coordinate multiple visits in a busy clinic setting. The care pathway aimed to incorporate strategies of preparing patients ahead of time [23, 38], and defining team responsibilities [19, 39], to streamline ACP. However, the COVID-19 pandemic introduced new time and resource pressures. A pivot to virtual care occurred organically in response to the pandemic and led physicians to reflect on challenges and future opportunities within this modality. Studies of ACP via remote consultations are emerging; an ACP intervention via videoconferencing was acceptable to persons with mild dementia [40]. Additional research may be necessary to develop recommendations of best practices for ACP in the context of virtual care. It will be important to assess the impact of virtual care on time pressure and workload for clinicians.

Regarding implementation and scale-up, clinicians referred to idiosyncratic issues within their existing practice, which illustrated how the care pathway fits within the current health care system. Compatibility between the work introduced by the pathway and the provincial context such as existing billing codes and documentation templates should be taken into account; Canadian primary care clinicians have previously described a need for remuneration and policy support for ACP [22]. 

Clinicians appraised the pathway as useful and impactful (NPT construct: reflexive monitoring). The stepped process and tools prepared patients and helped them feel more confident to have ACP conversations. More in-depth conversations, which guarded patient safety and comfort, further promoted decision-making confidence for all parties. Confidence and strong communication skills can in turn enable ACP uptake [20]. These findings support that the pathway structure facilitates meaningful discussions with patients [41]. Positive interactions bolster patient-provider trust, potentially mitigating barriers where patients fear negatively affecting their relationship with their physician if they discuss ACP [34]. This level of impact furthermore emphasizes the importance of talking about patients’ values and wishes for care when the patient is relatively well, so that the pathway can be revisited as the patient’s health status changes [8]. In light of this, adaptations of the pathway should accommodate patients who revisit ACP after initial conversations, in addition to patients who are newly-introduced to ACP. Additionally, an alert or trigger in the EMR for patients who meet certain criteria, e.g. related to age or illness, can keep clinicians alert to the need for ACP in patients at risk for health decline.

### Limitations/strengths

Strengths of this study include the detailed feedback about clinician experiences generated by the semi-structured interview and focus group format. There are also limitations to this study. First, although we include statements of impact on patients and SDMs, these statements were reported from the clinician perspective, not from the perspective of patients and SDMs themselves, and should be interpreted with this in mind. Second, the patients who participated in the pathway may have been those more amenable to ACP, and some clinicians reported buy-in issues for patients who were less amenable. Further reflection is needed on how to reach these patients and engage them in the first steps of ACP. Third, although physicians in BC who followed the SICG training but did not refer patients to the pathway were eligible to be interviewed, none participated in interviews. This may leave barriers related to participating in the project underexplored. However, qualitative findings also pointed to difficulties encountered by clinicians who did refer patients; these difficulties were mainly related to patients not being ready for conversations about serious illness and the end of life. It is possible that clinicians who could not refer any patients encountered similar barriers. Finally, the sample for this study was comparatively small, which limits its transferability. A broader range of experiences with implementing the pathway could be obtained through a larger sample across more clinics, and contribute to a fuller understanding of implementation and potential for integration into practice.

## Conclusions

This qualitative study contributes to our understanding of clinician experiences implementing an ACP pathway intervention by examining several different contexts: the Alberta and BC longitudinal outpatient generalist settings, including primary care settings. Results suggest that while the intervention may be implemented slightly differently in these contexts, core experiences with the pathway were that implementation into, and integration with, current practice were feasible. Across settings, similar themes recurred regarding usefulness of the pathway structure and its tools, impact on clinician confidence and interactions with patients, teamwork and task delegation, compatibility with existing workflow, and patient preparation and readiness. Clinicians were supportive of ACP overall and of the pathway in particular. While the pathway was implemented in a protocolized manner and thus did not overhaul clinical practice, clinicians’ experiences, suggestions for tailoring, and reflections on sustainability of the intervention offer valuable recommendations to consider when adapting the pathway for future implementation in primary care.

### Electronic supplementary material

Below is the link to the electronic supplementary material.

Supplementary Material 1

Supplementary Material 2

Supplementary Material 3

Supplementary Material 4

Supplementary Material 5

## Data Availability

The datasets analyzed during the current study are not publicly available; participants did not consent for data to be shared beyond the study.

## Abbreviations

ACP: Advance care planning
AD: Advance directive
BC: British Columbia
SDM: Substitute decision maker
SICG: Serious Illness Conversation Guide
