# Supplementary material for: Clinicians’ experiences implementing an advance care planning pathway in two Canadian provinces: a qualitative study
Source: BMC Prim Care. 2024 Jun 15;25:217. doi: 10.1186/s12875-024-02468-4 (PMC11179357; doi:10.1186/s12875-024-02468-4)
Supplement: Supplementary file 2 — Supplementary Material 2 [file 12875_2024_2468_MOESM2_ESM.pdf]

Additional File 2. ACP Pathway steps

The ACP pathway is an intervention based on the Serious Illness Conversation Guide (SICG).[1] Participating physicians and Allied health professionals received one 2.5 hour SICG training, conducted by members of the project team (AT, DB) who previously attended SICG Trainer-the-Trainer workshops.

The patient-facing portion of the intervention consists of three steps:

Step 1. The first step begins with completing informed consent, and completing the research questionnaires as part of the study procedures. Then, patients receive information about the general process of ACP, and about the substitute decision maker (SDM). Patients are encouraged to identify a potential SDM for the next step. Last, patients receive a province-specific ACP workbook: the Conversations Matter workbook in Alberta,[2] or the ACP resources from the BC Centre for Palliative Care.

Step 2. Step two is an ACP education and values clarification session. In this session, Allied health professionals (social workers, registered nurses) confirmed the patient's choice of a SDM. After this, the Allied health professional uses the *Explore* section of the SICG, and facilitates completion of the End of Life Values Best-Worst Scenario Online Tool with the patient.

After an introduction and an "about me" section, which includes questions such as the patient's age, the Best-Worst Scenario Online Tool introduces sets of three issues for the patient to rank from most to least important, when considering medical treatments they might want in the case of serious or life-threatening illness. Based on the patient's responses, the tool created a summary chart of what matters most to the patient, e.g., living as long as possible or avoiding the use of machines to keep the patient alive. The Allied health professional then reviews the summary chart together with the patient.

This chart is added to the patient's Dear Doctor letter, which alerts the patient's physician that the patient had an ACP pathway visit and indicates a wish to review the patient's preferences. The allied health professional conducting the pathway step provides a copy of the letter to the patient's physician, and makes a copy for the patient to keep (e.g. in the "Green Sleeve" for patients in Alberta).

In BC, steps 1 and 2 were combined into one visit with the research coordinator and a research nurse. In Alberta, step 2 was scheduled with an allied health professional during a second visit 2-6 weeks after step 1.

Step 3. In preparation for step 3, the physician reviews the Dear Doctor letter, which can be used during the Step 3 visit to confirm the patient's goals and trade-offs for care. Approximately 2-4 months after the first visit, patients meet with the physician for step 3, which focuses on finalizing and documenting ACP. During the visit, the *Assess, Share*, and *Close* sections of the SICG structure the conversation finalizing patient goals and wishes. These are documented using existing means in each province, e.g. the patient electronic medical record; the Alberta Health Services "Green Sleeve" containing ACP forms.[3]

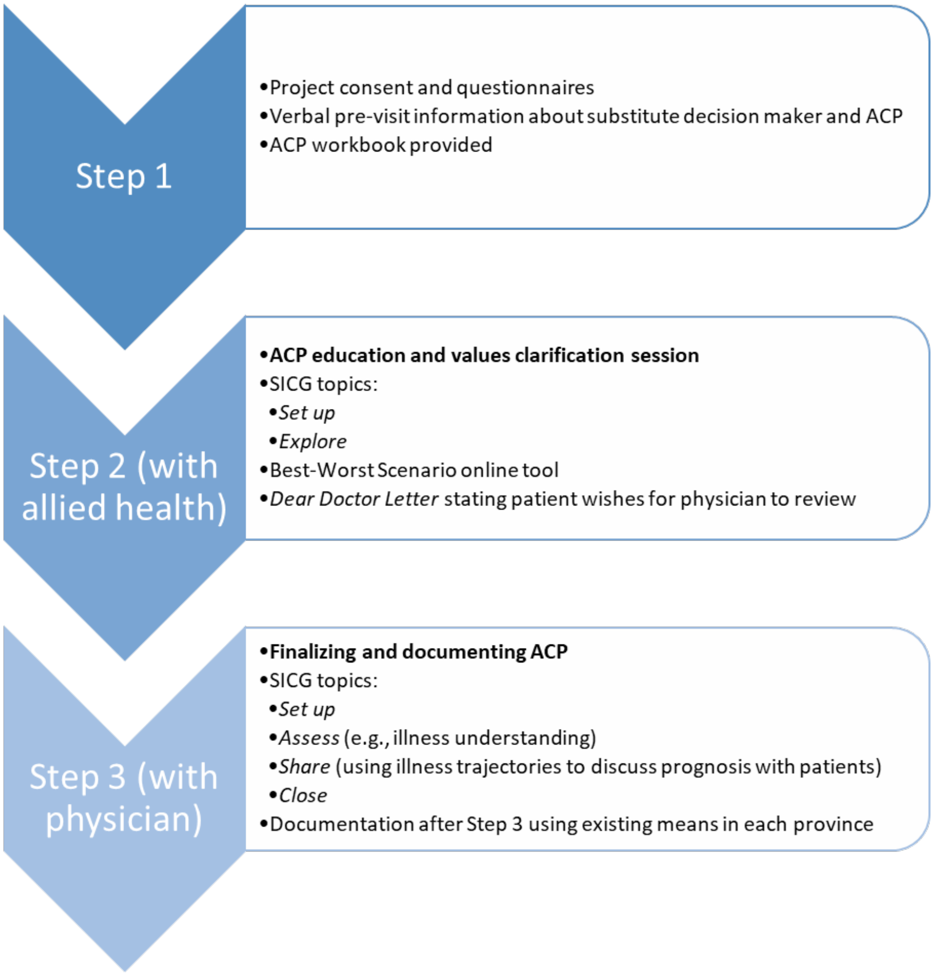

Figure 1. ACP pathway steps

References

1. Bernacki RE, Block SD, Force for the AC of PHVCT. Communication About Serious Illness Care Goals: A Review and Synthesis of Best Practices. *JAMA Intern Med*. 2014;174(12):1994-2003. doi:10.1001/jamainternmed.2014.5271

2. Alberta Health Services. Conversations Matter: Planning for your future healthcare. 2019. https://myhealth.alberta.ca/Alberta/AlbertaDocuments/conversations-matter-guide-english.pdf.

3. Palliative and End of Life Care Team. Green Sleeve. MyHealth.Alberta.ca. https://myhealth.alberta.ca/health/Pages/green-sleeve.aspx?hwid=TFiJW8-S9c4. Published 2018.

ACP pathway visits: Alberta

ICAN-ACP- Family Medicine/Primary Care

Visit One: Consent and ACP Intervention with Research Assistant

Consent process:

"You are here today because Dr. X suggested that you might be able to benefit from this study about advance care planning. Advance care planning is a process in which people talk about their values and preferences for future medical treatment with their family +/- loved ones and health care provider so that everyone is aware of their choices in advance. Through this process, you will choose an individual in your life who will be a part of an ongoing conversation with you regarding your values and beliefs so that they can make health care decisions for you in the future in the event that you are not able to make those decisions yourself. This person is called a substitute decision-maker. This study will help you along with the process of choosing your substitute decision maker, learning about your own wishes, and communicating them. Completing advance care planning will likely reduce your stress and anxiety and that experienced by your family members when you become seriously ill and have to make important treatment decisions regarding your care. The purpose of the study is to evaluate if our process helps patients better know their wishes and communicate them to others.

Before we begin though, you need to understand what is involved in the process. Basically, you will be asked to attend three appointments with us - this visit being the first one. This visit will include an overview of advance care planning, why it is important, and a lesson on how best to choose your surrogate-decision maker. During your next visit, we will have a detailed conversation about your values and beliefs regarding future medical care, and we will document them for you. Finally, your last visit will be with your doctor, where he/she will put the values and beliefs that we discussed in that second visit into the context of your illness so that they will officially be reflected in your future medical care.

We will ask you to try to bring a person who is your substitute decision-maker to the last appointment- the one with your doctor. if at all possible. Finally I would like to emphasize that you are absolutely not obligated to participate in this study, it is entirely voluntary. You can talk to your doctor about advance care planning whether or not you are a part of the study, and choosing not to participate would in no way affect your relationship with your doctor, or the care or treatment you receive.

Do you think you are interested at this point?

[If yes] I have here a consent form that outlines everything I just told you in greater detail, and I encourage you to read through that right now to be sure you understand the process. If you have any questions at any point, feel free to ask. Then, if you decide you want to participate, I ask that you write your name here, and sign here [mark X where they should sign].

Demographics and Baseline Measures:

"Okay, first I have a survey for you regarding some demographic and health related information that I would love for you to fill out for me. We use this information for analysis purposes - this information will not be connected to your name to anyone outside of the study team. I encourage you to answer all the questions, but please remember that you are not obligated to answer if you don't feel comfortable."

Baseline ACP Knowledge:

"Advance Care Planning is a process that helps us ensure your future health care aligns with your values and wishes. If there is a time in the future where you cannot speak for yourself, it is important that your loved ones understand your wishes and are able to communicate these to your healthcare team. This process may bring peace of mind to you and your loved ones. I am going to show you a video that explains this process in more detail."

[video]- White board video from Speak Up

"Now that we've talked about the general process of Advance Care Planning, let's go through the process of choosing your surrogate decision-maker. As you know from the video, choosing a surrogate decision-maker is an important decision as this person will make health care decisions on your behalf in a situation where you cannot make these decisions yourself. There are a few things you should keep in mind when choosing your surrogate decision-maker.

First, this person can be anyone in your life over the age of 18: a family member or a close friend. It may be tempting to choose more than one person, such as all of your children if you have more than one, however it is important to keep it to one individual to avoid conflict between decision-makers.

Second, this person should be someone you trust. You will need to have a conversation with them so that they understand your own values and wishes, and you will need to trust them to make healthcare decisions **for you** based on your values and wishes, rather than their own or what they may "think" you want.

This person should also be confident and able to speak up on your behalf, and confident in making important and sometimes difficult health care decisions in stressful situations. They should also be able to speak and communicate your wishes clearly. Health care providers are required to offer you, or your substitute decision maker if you are not capable, detailed explanations of any investigations/treatments and their risks, benefits, and side effects. They are also required to answer any questions before consent is received. It is important that your substitute decision maker is confident and capable of asking questions and weighing these risks and benefits accordingly.

Finally, ensure that your potential surrogate decision-maker is available and willing to take on this role - they should be fully prepared to speak on your behalf, and should be a large enough part of your life that you would feel comfortable talking to them about these things in an ongoing process. It is important to realize that health conditions are complex, and it is not possible to discuss or anticipate everything that may happen. This

is why it is important to continue the conversation about what's important in life for you, with your substitute decision maker beyond what you do for this study.

Please take some time to consider these criteria and think about someone in your life who might be available to fill this role. We will need you to identify this individual in your next meeting with us, and we ask that you bring him or her with you to your third ACP visit with your doctor. Do you have any questions about this process?"

Workbook- Conversations Matter Book

"Finally, I just wanted to show you this booklet that will be going home with you. This outlines everything we covered today in greater detail, including how to choose a surrogate decision maker. Here at the back, there is a checklist that lays out everything that we aim to have done by the end of this study. We want to make sure that you will confidently be able to check each of these off, and be happy with the plan that we have set for you. Does this make sense?

SICG Patient Prep Letter

We also have this letter for you as well, which outlines the things that we will be covering both in your next meeting as well as the third visit with your doctor. If you could read through this and prepare for your visits by just thinking about the things in this letter, it will help us to have a more thorough and helpful conversation during your following visits. Is that okay with you?"

<u>TO DO's</u>

Consent Form

Fill out Paper Demographic Form

Give Patient Conversation Matters Book

Give Patient SICG Prep Letter

Follow-up Slip to Book 1 hr iCAN-ACP Visit 2 with RA/SW/RN

Visit Two: Patient & Allied Health Professional/Research Assistant

"Just to check in, did you get a chance to think through the Advance Care Planning material you went through with [research assistant] during your last visit?

How are you feeling about this process so far? Did you pick a surrogate decision-maker? Just to make sure, lets revisit the criteria to make sure you are happy with your choice.

- Are they over the age of 18?
- Do you trust them to make decisions for you based on what YOU would do?
- Are they confident and able to speak up on your behalf?
- Are they able to speak up in difficult and sometimes stressful situations?
- Can they communicate your wishes clearly?
- Are they available and willing to take on this role?

For today's meeting, we're going to be opening up the conversation a little bit, and looking at the bigger picture of your values and wishes while taking the emphasis off of your specific health condition. Thinking about and sharing your wishes will give you more control over the care you get. It will also help prepare your loved ones to make decisions for you if you can't make them at some point in the future. Knowing what you want can help ease the burden on your family of making hard decisions for you if you can't speak for yourself.

Talking about the future won't change the plans we have made so far about your treatment, unless, of course, you want to. We will keep providing the best possible care to control your illness in a manner that you wish.

We understand that your wishes may change over time - no official decisions are necessary today. This is the beginning of an ongoing conversation. We know that you may have other questions or concerns in the future. We will continue to support you and answer your questions so that you can make informed decisions.

Does this sound okay to you? Do you have any questions?

We will start off with a quick computer task used to help us determine your values and wishes regarding your future medical care. You will be presented with seven 'values' regarding your future health care that will outline certain aspects of care that you may or may not find important. Each step of this process will ask you to rank three of these values from most important to least important. You will only have to indicate the most important and the least important - please leave the value you rank in the middle empty.

Please don't feel pressured while you fill out this survey - your rankings are not set in stone. This is only a tool to help you think about these values and discuss them with me. Please read through the written instructions before you begin, and feel free to ask if you have any questions.

[Let patient fill out the survey]
[Print out 4 copies of the screenshot of their results]

Thank you for doing that. I would love to walk through these values with you to see if this resonates with you. [Give a print out to them, and walk through results with the patient - "does the [top value] resonate with you as being the most important to you?" "does the [bottom] resonate with you as being the least important to you?"]

[Because we will be testing these SICG questions, please try to ask these questions with this language and in this order]:

GOALS: If your health situation worsens, what are your most important goals? *Short-term vs. long-term goals?*

FEARS/WORRIES: What are your biggest fears and worries about the future with your health?
(*We will pilot this initially and make official decision after testing*)

STRENGTH: What gives you strength as you think about the future with your illness

FUNCTION: What abilities are so critical to your life that you can't imagine living without them?

TRADE-OFF's: If you were to become sicker, how much are you willing to go through for the possibility of gaining more time? (*Refer to results of Best-Worst Scenarios to help with this*)

FAMILY: How much does your family know about your priorities and wishes? (Suggest bring family/ or SDM to next visit to discuss together)

- *Provide Patient with Family Communication Guide to discuss with SDM at home and bring SDM to next app with MD.*

Finish meeting with the following:

[Summarize verbally key goals/ priorities with regards to values preferences (trade-offs), function and goals]

[Document conversation in Dear Dr. Letter and AHS Tracking Record while confirming everything with patient]

"I just want to quickly explain this document, called a Personal Directive. This is a legal document which your health care team will look at to determine who you have appointed to be your surrogate decision-maker. This document ensures that everything we discussed today will be reflected legally and carried out by your health care team. Today, if you feel comfortable with your decision about appointing [name of SDM] as your

surrogate decision-maker, we can write his/her name into this spot on the personal directive. Is that okay, or would you like to come back another day to complete this document?"

"For the next steps, I will be giving a copy of this letter to your doctor, and will make a copy to put in your Green sleeve as well. This Green sleeve should hold all materials with regards to Advance Care Planning such as this Personal Directive and the summary from today's visit. Your doctor will be adding another form to it at your next next visit. In between health care visits (anywhere- the clinic, the hospital, Emergency Room etc), the Green sleeve needs to be kept by your fridge so that everyone knows where it is, including EMS Paramedics if they were ever to need to be called to your home. Please make sure you bring your Green sleeve with you to your third visit with your doctor.

We would also like for you to talk to [name of SDM] about what we talked about today and review these documents in the Green Sleeve, and ask if they can come with you to your next appointment. Do we have permission from you to contact them in advance to make sure they consent to the study and understand their role?

[If yes..] Great, thank you. [Name], the research assistant, will contact [name of SDM] between now and then. It would help if you could tell [name of SDM] that they will be contacted about this, just to make sure they are expecting the call. Is that okay?

Great, would you like to book the next appointment right now? [If yes:] I can come with you to the front desk to help with booking that appointment. Don't forget to bring your Green sleeve with you to the next appointment!"

TO DO's:

1) Name and contact for SDM for [the research assistant] to contact
2) Fill out Visit 3 booking slip for 30 min ACP visit with MD (patient to attend with SDM if possible)
3) Print out Tracking Record started/recorded in EMR for Patient's Green sleeve ([Research assistant] to do this written on paper copy until this visit is handed to [research coordinator])
4) Fill out Personal Directive if possible and put in Green sleeve
5) Print out copies and label of Best Worst Scale Tool and copy Dear Dr. Letter to be scanned into pt's chart.
6) Put Best Worst Scale Tool printout and Dear Dr. Letter into Green sleeve.
7) Give Green sleeve to patient and remind to put by fridge and bring back to next ACP visit.

Visit Three: Patient, Surrogate Decision-Maker & Physician

iCAN ACP Advance Care Planning

1. **SUMMARIZE and CONFIRM:**

"I understand that you had a conversation with SW/RN/RA NAME X many weeks ago. I received a letter (with the Best Worst Scale Graph) summarizing your conversation with her. I would like to continue the conversation today to be able to discuss your specific health condition/illness with you and how we can work together to be able to help you achieve the goals that you outlined with NAME in the last visit. Would that be ok?"

"If so, we can start with confirming some of the information that was summarized from that conversation with NAME:"

- Confirm goals/ trade-off/critical abilities (as per Dear Dr. Letter and Tracking Record)
- Ask if spoke with SDM
- Any questions/ clarification of anything or changes needed upon time/reflective/discussion with SDM since?
- Does the SDM have any questions at this point?

"I'd like to talk about what is ahead with your illness and do some thinking in advance about what is important to you so that I can make sure we provide you with the care you want — is this okay?"

2. **ASSESS AND SHARE SPECIFIC HEALTH CONTEXT INCLUDING PROGNOSIS/ILLNESS TRAJECTORY (from SICG):**

ASSESS

- "What is your understanding now of where you are with your illness or health condition?"
- "How much information about what is likely to be ahead with your illness or health condition would you like from me?"

SHARE

- **"I want to share with you my understanding of where things are with your illness/health condition..."**

1. ***Uncertain***: "It can be difficult to predict what will happen with your illness. I hope you will continue to live well for a long time but I'm worried that you could get sick quickly, and I think it is important to prepare for that possibility."
2. ***OR Time:*** "I wish we were not in this situation, but I am worried that time may be as short as ___ (express as a range, e.g. days to weeks, weeks to months, months to a year)."
3. ***OR Function:*** "I hope that this is not the case, but I'm worried that this may be as strong as you will feel, and things are likely to get more difficult."

[USE Illness Trajectories Pictured Below in Training of MDs for Chronic Illness etc]

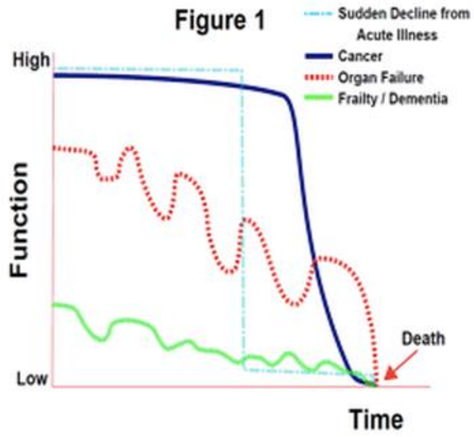

Image From:

Fast facts and concepts #326
Illness Trajectories: Description and Clinical use
Paige Comstock Barker, MD & Jennifer S. Scherer, MD

Based on work by: Murray et al. Illness Trajectories and Palliative Care. BMJ. 2005 Apr 30; 330(7498): 1007–1011.

3. <u>TRANSLATE GOALS/FEARS/WORRIES AND INDIVIDUAL HEALTH CONTEXT INTO AHS GOALS OF CARE DESIGNATION:</u>

-Circle back to goals/trade-offs/ fears-worries/function/ sources of strength from Dear Dr. Letter
-Summarize key goals/ priorities (Future medical decision goals to achieve in context of values and preferences)

- "I've heard you say that ___ is really important to you. Keeping that in mind, and what we know about your illness/health condition, I recommend that we _________________ [translate to Goals of Care Designation starting point] **to help us achieve what you want (and don't want) based on what has been discussed in these 2 ACP study visits**.
- "This will help us make sure that your treatment plans reflect what's important to you. How does this plan seem to you? How does this plan seem to you, the SDM? Any questions/ concerns? We will do everything we can to help you through this."

4. <u>DOCUMENT IN TRACKING RECORD AND GOALS OF CARE DESIGNATION ORDER FORM in VISIT NOTE IN EMR</u>

- **PRINT OUT TO PUT IN GREEN SLEEVE TO GIVE TO PATIENT TO PUT BY FRIDGE AT HOME (Along with Dr Doctor Letter and Personal Directive already in the Green Sleeve)**

iCAN ACP Advance Care Planning

Advance Care Planning Values and Preferences

Date: ______________________________

Dear Doctor ______________________ (name),

I participated in the Improving Advance Care Planning in Primary Care for Canadians (iCAN-ACP) program on: ________________________ (date) and wish to discuss/review with you my preferences for care for when I am seriously ill. I have met with the allied health professional / iCAN-ACP research assistant ________________ (name of RA/SW/RN) to go through the 'values clarification tools' that helped me think about and clarify what is important to me when considering treatments when I am seriously ill. The answers to the following questions show what is most important to me at this time:

[Print off Best Worst Scale (graph) results and include with letter]

My substitute decision-maker(s) is (if ready to make this legally binding, please complete name in Personal Directive):

Name	Relationship to me	Contact information (optional)

The most important goals if my health condition worsens are:

____

My fears/worries include:

____

What gives me strength in regard to the future of my illness include:

____

The abilities that are critical to me are:

____

We discussed the trade-offs that I'm willing to make to be:

____

Other issues I would like to discuss about this topic include:

____

____

____

____
