# Supplementary material for: Clinicians’ experiences implementing an advance care planning pathway in two Canadian provinces: a qualitative study
Source: BMC Prim Care. 2024 Jun 15;25:217. doi: 10.1186/s12875-024-02468-4 (PMC11179357; doi:10.1186/s12875-024-02468-4)
Supplement: Supplementary file 3 — Supplementary Material 3 [file 12875_2024_2468_MOESM3_ESM.pdf]

7-item, with values statements/risks/benefits table. 2

Screenshot 1: Introduction 2
Screenshot 2: About me, part 1 3
Screenshot 3: About me, part 2 3
Screenshot 4: About me, part 3 4
Screenshot 5: Values instructions and table 5
Screenshot 6: Values Questions 7
Screenshot 7: Summary 8

7-item, with values statements/risks/benefits table.

Screenshot 1: Introduction

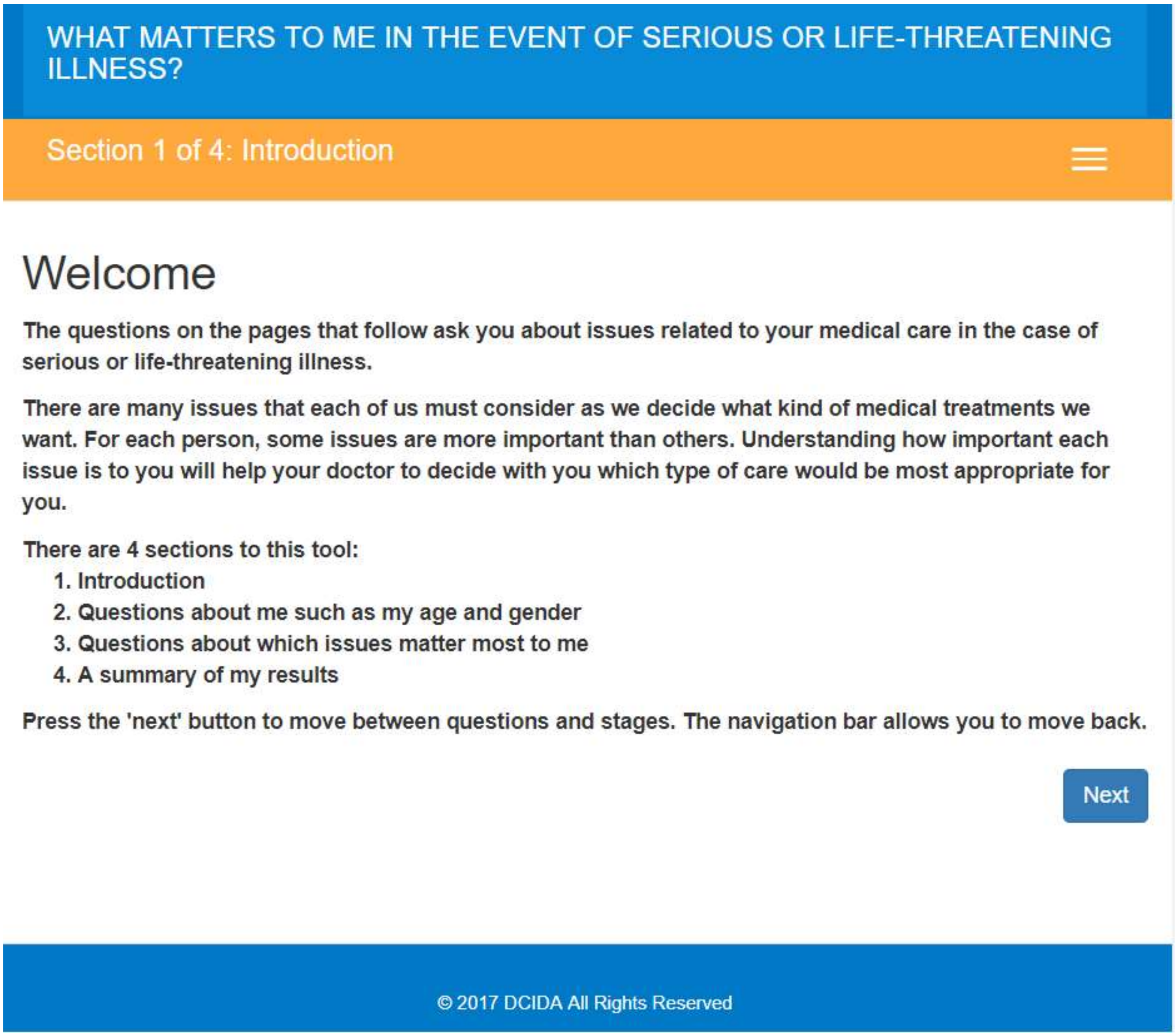

WHAT MATTERS TO ME IN THE EVENT OF SERIOUS OR LIFE-THREATENING ILLNESS?

Section 1 of 4: Introduction

Welcome

The questions on the pages that follow ask you about issues related to your medical care in the case of serious or life-threatening illness.

There are many issues that each of us must consider as we decide what kind of medical treatments we want. For each person, some issues are more important than others. Understanding how important each issue is to you will help your doctor to decide with you which type of care would be most appropriate for you.

There are 4 sections to this tool:

1. Introduction
2. Questions about me such as my age and gender
3. Questions about which issues matter most to me
4. A summary of my results

Press the 'next' button to move between questions and stages. The navigation bar allows you to move back.

Next

© 2017 DCIDA All Rights Reserved

Screenshot 2: About me, part 1

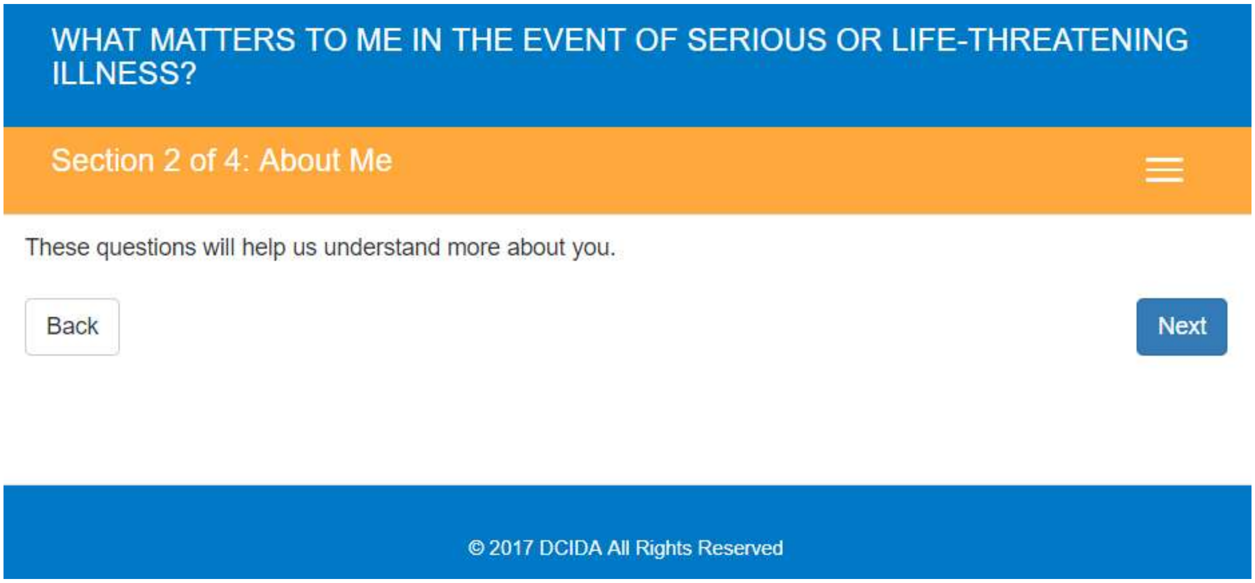

Screenshot 3: About me, part 2

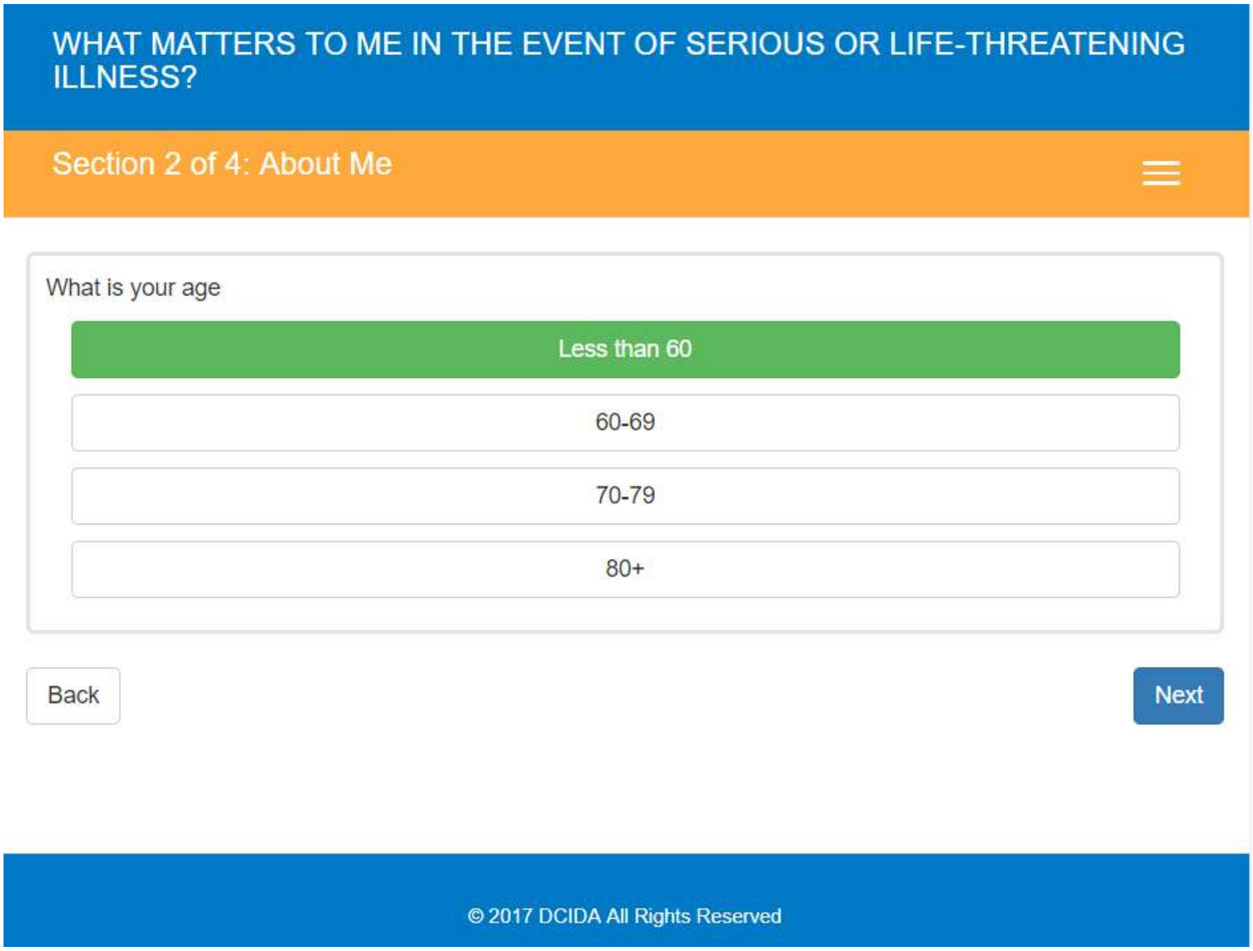

Screenshot 4: About me, part 3

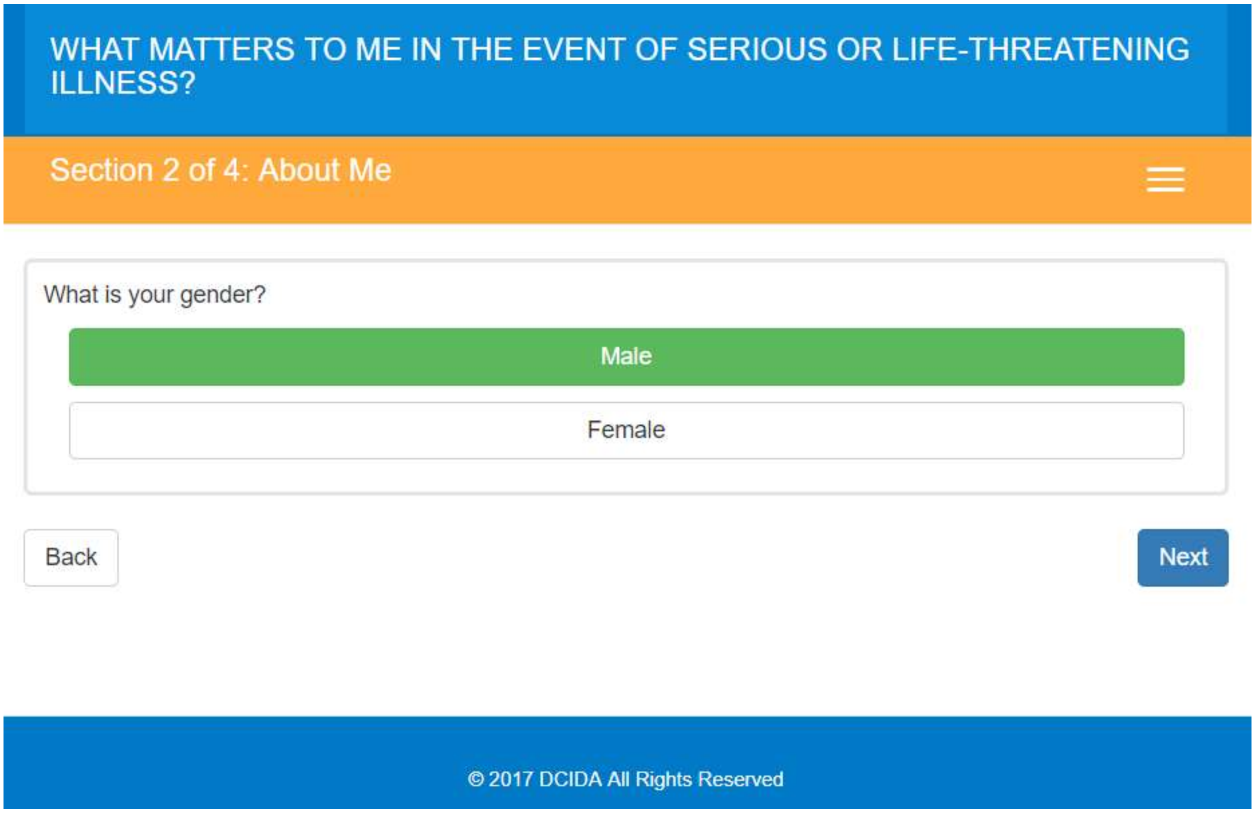

Screenshot 5: Values instructions and table

WHAT MATTERS TO ME IN THE EVENT OF SERIOUS OR LIFE-THREATENING ILLNESS?

Section 3 of 4: My Values

How to complete these questions

We will show you 7 sets of issues. Each set has 3 different issues.

What we want you to do is simple - please consider each of the issues and tell us:

- Which ONE of the 3 issues is **most** important to you when considering medical treatments you may want?
- Which ONE of the remaining 2 issues is the **least** important to you when considering medical treatments you may want?

There are no right or wrong answers - we are only interested in your opinions.

Issue	Benefit	Risk
1) **Live as long as possible**<br>I want to live as long as possible.	Living as long as possible	May require the use of life support machines
2) **Avoid Machines**<br>I want to avoid the use of machines in order to keep me alive.	Not being limited by machines and/or being in a hospital	May die from a severe illness or sudden death
3) **Lessen Symptoms**<br>I want to avoid symptoms such as pain and shortness of breath.	Being free of painful or uncomfortable symptoms	May not receive some types of medical treatment that could extend life but make symptoms worse (i.e. chemotherapy)

4) **Decision making & Independence**<br>I want to be able to make my own decisions about my care and live as independently as possible.	Medical goals are in line with your own wishes	Following medical goals may not improve health or result in a longer life
5) **Remain Active**<br>I want to continue to be able to participate in the activities I like to do.	Continue to be as active as possible	May not receive certain treatments that could extend life if it requires being bed-bound or under sedation
6) **Think Clearly**<br>I want to be able to think clearly and not be confused.	Maintaining your ability to carefully consider the situation at hand	May not be possible if disease state worsens (i.e. delirium); might experience pain if thinking clearly requires withholding pain medication
7) **Religious Beliefs**<br>I want treatment consistent with my religious and spiritual beliefs.	Decreased moral or spiritual distress	May conflict with recommended treatment of disease

Back

Next

© 2017 DCIDA All Rights Reserved

Screenshot 6: Values Questions

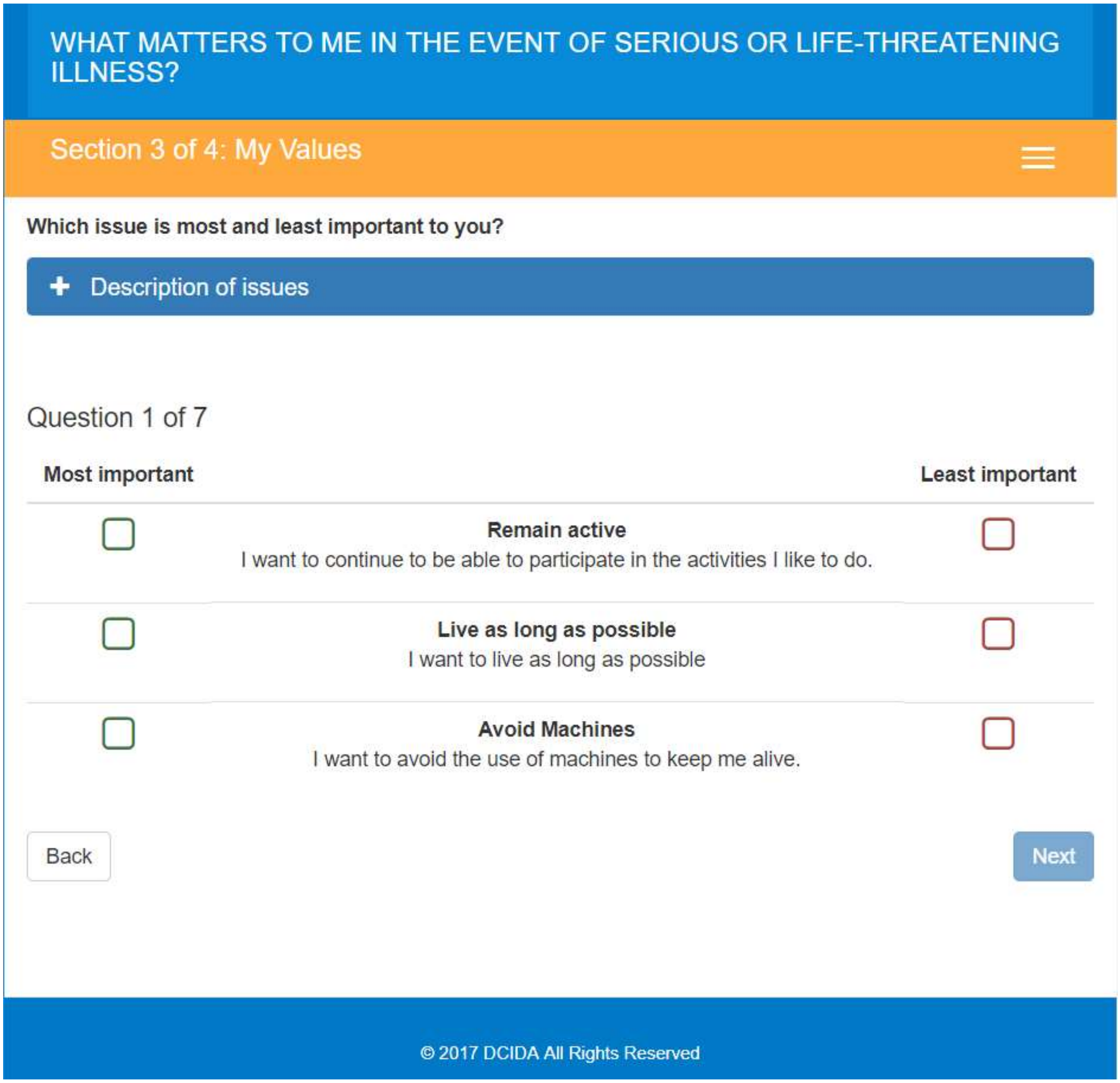

Note: "Description of issues" link opens to display table on previous screenshot.

Note: This screen repeats 7 times with different value combinations displayed. Only one example included here.

Screenshot 7: Summary

WHAT MATTERS TO ME IN THE EVENT OF SERIOUS OR LIFE-THREATENING ILLNESS?

Section 4 of 4: Summary

You have completed all the questions

This information will help you and your doctor to choose the most appropriate treatments for you if you have a serious or life-threatening illness.

The chart below describes what matters to you from most (top) to least (bottom)

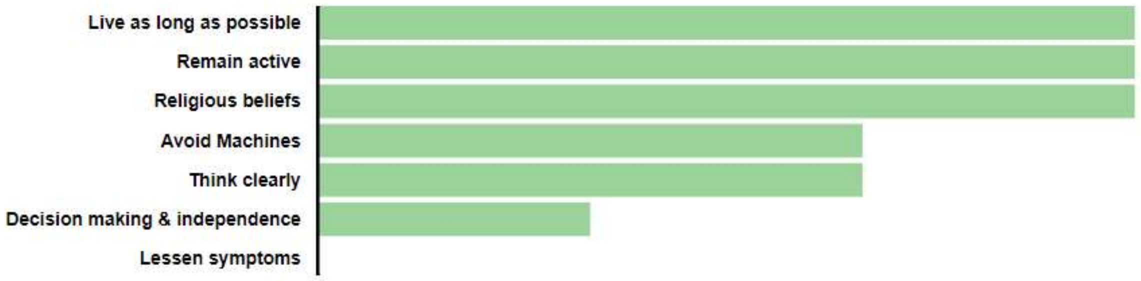

Back
