# Supplementary material for: Clinicians’ experiences implementing an advance care planning pathway in two Canadian provinces: a qualitative study
Source: BMC Prim Care. 2024 Jun 15;25:217. doi: 10.1186/s12875-024-02468-4 (PMC11179357; doi:10.1186/s12875-024-02468-4)
Supplement: Supplementary file 4 — Supplementary Material 4 [file 12875_2024_2468_MOESM4_ESM.pdf]

Pathway Implementation Evaluation

Overall Research Question- What is the lived experience with the pathway?

Draft Interview Guide

Introduction/Context of Questions

We would like to get some information from you about your experience with the care pathway. As a reminder, the care pathway we refer to includes some components developed to make ACP a structured process. Briefly, these are:

- A method to identify patients,
- A conversation guide for clinicians (nurse or social worker), used in combination with values clarification tools,
- Results of that conversation in *the Dear Doctor Letter* and the ACP Record,
- A conversation guide for physicians to follow up

To support the pathway, you were provided with a one-time training for the conversation guide.

Interview Questions

NPT generic question	Question wording	Prompts
**Background**	1. Please describe your role in the care pathway and how much experience you have had with it to date.	
**How did the intervention effect the work of the practice?**	2. How is the care pathway similar or different from how you previously approached Advance Care Planning discussions with patients?	• Patient identification/prompting<br>• Provision of Structure to conversation<br>• Tools (information provision, values clarification, documentation)<br>• Communication training<br>• Preparation of patient<br>• Allied health involvement
	3. What if anything changed about the way physicians/clinicians work together when using the care pathway?<br>Whose roles changed and how?	• Role clarification<br>• Scope of practice<br>• Communication<br>• Collaboration

NPT generic question	Question wording	Prompts
**How compatible was the intervention with current work processes?**	4. How compatible was the care pathway with your current work processes?<br>How was your typical work flow impacted by the care pathway?	• Patient identification/prompting<br>• Provision of Structure to conversation<br>• Tools (information provision, values clarification, documentation)<br>• Communication training<br>• Preparation of patient<br>• Allied health involvement
**Did it promote or impede work?**	5. Tell me about feasibility and workload issues -<br>What were the barriers / facilitators to using this pathway?<br>What would you change?	• Prioritizing against other things<br>• Confidence/self-efficacy to conduct ACP<br>• Willingness to conduct ACP/follow pathway<br>• Supports of the pathway to conducting ACP
	6. How did the pathway affect the time taken or efficiency of your visits?	• If time/efficiency affected, was the time 'well spent'?
**What effect did it have on consultations?**	7. Compared to how you previously approached ACP discussions, how did the care pathway affect your ACP conversations with patients and their SDM/family member(s)?	• (Perceived) patient comfort/experience<br>• Outcomes
	8. Thinking about the components of the pathway -<br>Are they useful/not useful?<br>Which do you believe are essential?	• Patient identification<br>• Conversation guide<br>• Structured approach, patient preparation<br>• Dear Dr. Letter<br>• Integration of [Health Authority] ACP Record
**What is perception of intervention at this point?**	9. Did people seem supportive of the pathway - why or why not?	• Physicians/Clinicians<br>• Patients/SDM/Family
	10. What benefits did you perceive by using this pathway approach?<br>And conversely, what were the challenges?<br>Were there any unintended effects?	
	11. What would help this care pathway continue (if beneficial)?<br>What would hinder it from continuing?<br>What would improve it?	
