# Supplementary material for: Clinicians’ experiences implementing an advance care planning pathway in two Canadian provinces: a qualitative study
Source: BMC Prim Care. 2024 Jun 15;25:217. doi: 10.1186/s12875-024-02468-4 (PMC11179357; doi:10.1186/s12875-024-02468-4)
Supplement: Supplementary file 5 — Supplementary Material 5 [file 12875_2024_2468_MOESM5_ESM.docx]

**Additional File 5: Table S1**

**Table S1. Domains, themes, and subthemes with illustrative quotes**

| Domain | Theme | Subtheme | Illustrative quote |
| --- | --- | --- | --- |
| Care Pathway | Facilitators | Documents, forms, and tools are helpful | “As you get comfortable with these meetings, then the summarized version of the conversation I think is a helpful guide. The Dear Doctor letter I think is quite imperative and I use it even if I get a standard referral that’s not part of this study […]. “ (Alberta, social worker) |
|  |  | Sequential structure is easy to implement | “I think having the pathway, you know, there’s steps and there’s a starting point and an end point. There’s things to be achieved along the way. That’s a little more structured and what I would’ve done before, so that’s a benefit.” (Alberta, physician) |
|  | Barriers | Appointments: preparation, duration, and modality | “Like I know that the patient has done this, documents have been shared, this has been completed, the letter’s there, it’s in the chart. Now the patient’s coming to me. Sometimes it just seemed to be a bit complicated to get everything together in time for the visit.” (Alberta, physician) |
|  |  | Negative patient experiences with the pathway | “Like I said, I feel like I had the worst case example in terms of patient engagement because her decision-maker didn’t want to be part of the discussion, she didn’t want to do the best and worst list, she didn’t want to do the Dear Doctor letter […]” (Alberta, physician) |
|  |  | Clinical care coordination | “Your Dear Doctor letter was roughly[…]the <health authority> template and it included all those things […].  Then we pulled as much out of it as possible to make it something that would, would suit the needs of the health authority and even that wasn’t enough because they would only accept the official branded ACP record […]” (BC, physician) |
|  | Future sustainability | Adapt to emerging needs (Virtual care, COVID, …) | “This pandemic has changed a whole lot of things for everybody and so [...] I think the pathway will have to be adapted to meet the needs of virtual care and phone care.” (BC, physician) |
|  |  | Embed and normalize ACP | “Where if it became an organic thing that everyone in the clinic it’s one of the […] questions that are asked, then it becomes something that’s more normalized, which it should be.” (Alberta, physician) |
|  |  | Expand training | “I actually think it’s one of those things which would be good to have a refresher on […] every so often too, like every other year or something like that, because it’s something that we don’t always keep top of mind with everybody.” (BC, physician) |
|  |  | Need for communication and bridging tools | “…some more communication between the clinician and the physician because I could see that there might be some things that were shared that maybe didn’t come up with the physician, so I don’t know if there is some other mechanism aside from the Dear Doctor [letter] […]” (BC, general internist) |
|  |  | Broader health care system implications | “[…] I think knowing where to put this pathway to start would be really important, I’m just trying to think about what the streams or referrals would be and I would think that family practice is a great place to start.  I think the internal medical clinics that […] see a lot of the discharges from hospitals is a good place, the oncology ward for obvious reasons.  And then I think the next level would belong to some specialty clinics, cardiology, gastroenterology, respiratory.” (BC, general internist) |
| Clinician impact | | | |
| Clinical Practice | Facilitators | Patient/SDM willingness, readiness, and preparation | “[…] I think sometimes I would get these kinds of referrals in times past to talk about advance care planning and there would be, you know, maybe somewhat frequent times where the patient might say, “I really don’t know exactly why I’m here”.” (Alberta, social worker) |
|  |  | Positive impact on clinicians | “I think I'm more confident in the decision-making approach, because of everything that came ahead of the patient coming in to see me.”  (Alberta, physician) |
|  |  | Positive impact on clinicians’ interaction with patients | “So I think the preparation allowed people to comfortably talk about these things. Of course, there was tears at times but I think that, you know, having a clinician have a meeting with patients to talk about these things, whether it’s a nurse or a social worker or another type of clinician is helpful because we can be gentle in that conversation. So I felt everyone was safe doing it.” (Alberta, social worker) |
|  | Barriers | Practical challenges to visits | “[…] I think I shared that with you, how it just came at a crazy time where people were shut in, there was a lot of fear among [...] the population anyway, so […] it just was sort of a little bit harder timing to have that call, conversation.” (BC, physician) |
|  |  | Patients/SDMs may not be ready | “I didn’t find myself able to apply [the pathway] directly with well patients that frequently and I had a number of patients who […] probably weren’t quite ready to have some of those types of discussions regardless of the framing even though, you know, I would have considered them to be in the category where they probably would have benefitted.” (BC, physician) |
|  | Future sustainability | No billing codes | "I mean now in order to do some billings, you have to have an advanced care like that now so our complex care billing [is] generally tied in or if there was a billing for that. […] There’s no real billing for having that discussion, there is a billing for congestive heart failure, that visit, there’s a billing for but for a teleconversation it’s not specifically a billing for that. […] So it doesn’t really fall under counseling visit, so I think GP’s struggle […]”(BC, physician) |
| Teamwork | Facilitators | Social worker has the necessary skills for ACP and is a referral for more complicated conversations | “I'm more likely to refer to social work if it seems that there’s maybe like a more complicated discussion that has to happen or if a patient really just seems like wanting to maybe engage, but really just seems very on the fence about things or on the fence about who their substitute decision-maker should be. Like sometimes I think I'm referring to <the social worker> a little bit more, like I kind of know that she has that skill set now.” (Alberta, physician) |
|  |  | Awareness of staff | “Definitely I think our staff like here at the clinic were supportive and, you know, often would be like, “You need this paperwork” and come drop it off for me, so it was like really helpful that way.” (Alberta, physician) |
|  |  | Promotes teamwork and strengthens existing collaborative relationships | “Now nurse [name] can take the first thirty minutes of that, review, you know, the areas that she’s been trained to review over the serious illness conversation and then after, you know, twenty or thirty minutes I can focus on the areas that I wanted to address plus allude to the serious illness conversation, the best care plans that have been documented and just ensure that’s consistent with, you know, what [health authority] would need to know.” (BC, physician) |
|  | Barriers | Availability of resources : staffing, structural barriers, team composition | “I mean for most private physicians’ practices, you know, […] many of us don’t really have allied health working with us that much except for our front desk staff.  I, I mean in our, in our office we are lucky that we get, get an RN sort of into our practice within the past year and a half but incorporating her as a formal part of our sort of ACP process hasn’t really happened yet […]” (BC, physician) |
|  |  | Unclear division of tasks | “What happened in my case is that it wasn’t clearly labelled in the EMR that were [project] patients to start with, and so, they were assigned to residents, and I understood that we were supposed to do these interviews. So, that became quite problematic because we’re scraping off an hour throughout the day in which the residents aren’t just sort of … So I actually tried at one point to get the residents to watch me and to give feedback; that didn’t work either, so.” (Alberta, physician) |
|  |  | Problems coordinating between visits | “I almost wish that there was more built-in communication or shared appointment between me, the patient and [the social worker] because I almost felt like the patient was the go-between between me and [the social worker] […]” (Alberta, physician) |
|  |  | Different service models do not support the pathway in its current form | “I would say that typically in our clinic we don’t have allied health professionals, we work solo [. . . ] So in the hospital we have lots of allied health assistance but in the clinic we’re not used to really having anybody else there so the fact that somebody, that a new role is there, it’s just completely novel […], not a change or I don’t change the way that I see them or the way that their roles are. It’s just a completely new role that wasn’t there before.” (BC, physician) |
|  | Future sustainability | Training other staff | “And maybe, maybe as we move forward the nurses who participate in this manner should have more formal training and do it as has been done in the study.” (BC, physician) |
|  |  | Who will be available after the study is over? | “So I think the worry that I have in terms of like work planning moving forward is that [the social worker] might not have the capacity to do this for all of our patients and that we might need to think more broadly about how we’re going to do this like moving forward because she’s pretty busy.” (Alberta, physician) |
|  |  | Expanding visit 2 | “The other question I would have is, could the allied health worker actually go a little further along the path, in other words go to the point where they even talk about which level of care. And then, if the [physician] needs to be involved it’s actually fairly quick. Just the pathway, this way it’s left up to the physician.” (Alberta, physician) |
| Work processes | Facilitators | Efficiency and integration with current workflow | “Most of the time I’d see the patient for their follow-up visit and then they were contacted separately so it didn’t really actually affect my work flow at all, because that was done almost after I’d just talked to them at the end of the appointment about it, made the referral and then that was all done separately so it didn’t affect my work flow at all.” (Alberta, physician) |
|  |  | Recognition of inherent value of the intervention | “Yeah, definitely time well spent. I think these are important discussions that I often neglect to or forget to have. It’s just sort of one of many things that we try and do and often, unless it’s sort of you’re confronted with it, I either forget or I don’t do it. And I think actually this study was nice in that it brought it to the forefront for me and reminded me and my residents that it needed to be done.” (Alberta, physician) |
|  |  | Benefits of recruitment strategy | “It almost seems less threatening in some ways right, like, “We just put your name into a computer list and yours – or we put criteria into a computer list and your name popped up”, so it’s not like somebody went, “You need this ACP”. Like I feel that that as an element of like fear-inducing and that kind of more neutral, “Computer popped your name up and we’re calling you, would you like to come talk about this”.” (Alberta, physician) |
|  | Barriers | Individual/practice-level barriers | “Just that I had to know in advance which patient I would see that day, refer on did require a little bit of planning and giving the patient a head’s up about that kind of conversation as well.  So, you know, instead of just going through my regular clinic and then identifying them in the moment and referring them on I had to, had to have a little bit of preplanning.” (BC, physician) |
|  |  | System-level barriers | “I think that its, its challenging, its challenging in sort of the people, service worlds we work in to make large changes in how we do things quickly even with sort of the availability of certain types of videos to help people with called complex care planning.” (BC, physician) |
|  | Future sustainability | Virtual vs. in-person visits for different purposes and populations | “And then that’s why some of the ACP stuff sometimes falls to the wayside a little bit.  I think, I think phone visits could work for a set of patients who are, don’t have significant dementia or memory issues or hearing issues, I think there is a set of patients who would do fine on the phone thinking about that stuff. And then virtual visits I mean there’s technical challenges with those for sure with the older population (…)” (BC, physician) |
|  |  | Placing the pathway in context of health care system changes | “And the second thing is I think that the upcoming sort of changes to potential physician contracts for salary sort of physician may actually make a big difference here too in our ability to be able to offer some of the deeper stuff.” (BC, physician) |
|  |  | Tailoring to local context and adapting to individual clinic needs | “And I think it’s more the access and the availability I can see like in some, […] in a busy clinic and environment you’d just have somebody available and you can kind of have like a longer visit, whether its remotely or in person, and that just be included as part of the like a complex visit, that I could see where it would be helpful.” (BC, general internist) |
| Patient impact | | | |
| Preparation | Facilitators | Pre-work for follow-up visits | “I can tell you they got more invested in the process once they've met with [the social worker] and done that. Versus us just kind of like more passively handing them something and saying “Go ahead and rebuild this on your own.” (Alberta, physician) |
|  |  | Patient-centered, ongoing conversation | “And some of the patients, I think the more people that talk to them about advance care planning the more they get comfortable with it so it may not, this may be a first conversation or it may be a subsequent conversation. And so it’s hopefully building on things that they’ve already heard and whether they were in hospital before.” (BC, general internist) |
|  | Barriers | Patient lack of buy-in | “Yeah. And the three visits were one thing that they raised problem, their concerns about. So I just said, “Look. Don’t do it for me. We’re doing this for you.” And so a couple dropped out.” (Alberta, physician) |
|  |  | Need for more clarity, time, discussion | “If there’s any lack of clarity and still thinking and further discussion that needs to occur then that often needs to occur outside the clinic somewhere. So, if that’s obvious then it’s like “Hey, you know what? We need go in a little bit more and think about this.”” (Alberta, physician) |
|  |  | Difficulty translating goals into levels of care | “The focus of my discussion was definitely about understanding what the Goals of Care were, what the Goals of Care designations were. Without providing any direction and trying to translate into what the discussions that it had before. And I do think that made things easier, but that discussion still becomes technical. And it’s probably one of the more – It is important, but I think it’s also translating what patients want into a number and a letter, with an understanding of what that is. And the consequences of that, or the potential consequences, that’s challenging.” (Alberta, physician) |
| Readiness | Facilitators | Promotes readiness to have goals-of-care discussions and complete documents | “I think the benefit was that more people in our clinic, more patients in our clinic are completing their goals of care because of this.“ (Alberta, social worker) |
|  |  | SDM is aware of patient values and wishes | “I think it prepares the substitute decision-maker for the role and it also provides a lot of comfort for the patient that if their health changes and they don’t have the capacity to make their health-related decisions, that they have somebody to do that for them, that understands their role is to speak on their behalf.” (Alberta, social worker) |
|  | Barriers | Difficult transition from thinking about values to documenting goals of care | “Even though it did fit with their values, they just weren’t ready to believe that, and so their goals of care actually don’t reflect what their values and wishes are because they couldn’t get there despite our multiple attempts of trying to clarify it.” (Alberta, physician) |
|  |  | Lack of patient comfort and energy | “Where I do worry a bit is the flow for the patient. Instead of coming in to see a doctor for an hour, which is already a very long time for a patient, they may be there for two plus hours, a whole afternoon for them.  So I do worry about their comfort, their energy level, um, when, when these conversations are, are going on for a long time.”  (BC, physician) |
